# The growth and composition of primary and community-based care services. Metrics and evidence from the Italian National Health Service

**DOI:** 10.1186/1472-6963-12-393

**Published:** 2012-11-13

**Authors:** Francesco Longo, Domenico Salvatore, Stefano Tasselli

**Affiliations:** 1 Department of Institutional Analysis and Public Management, Università Bocconi, Milan, Italy; 2 CERGAS (Centre for Research on Health and Social Care Management), Università Bocconi, Milan, Italy; 3 Department of Management, Università ‘Parthenope’, Naples, Italy; 4 Fondazione SDN, Naples, Italy; 5 Judge Business School, University of Cambridge, Cambridge, UK

**Keywords:** Primary and Community based-care services, Italian National Health Service, Local Health Authority, Health expenditure

## Abstract

**Background:**

Over the past few decades, in OECD countries there has been a general growing trend in the prevalence of out-of-hospital healthcare services, but there is a general lack of data on the use of these services.

**Methods:**

We defined a list of 303 indicators related to primary and community healthcare services in collaboration with 13 Italian Local Health Authorities (LHAs). Then, for each LHA, we collected and analyzed these indicators for two different years (2003 and 2007).

**Results:**

Out-of-hospital care absorbs 56% of all costs in our sample of LHAs. Expenditure on outpatients’ visits to specialists and on diagnostic examinations accounts for 13% of the costs, while spending on primary care (including prevention and public health) accounts for 9%, and for intermediate structures (including those related to rehabilitation, elderly people, disabled people, and mental health) the figure is 11%. Different Italian LHAs have made different strategic choices with respect to primary and community-based care (PCC).

**Conclusions:**

Two distinct strategic orientations in the adoption of PCC services by LHAs has emerged from our study. The first has been an investment mainly in ambulatory and home-based primary care services in order to increase the number of low-complexity settings. A second strategy has prioritized the allocation of resources to intermediate inpatient structures for specific types of patients, namely elderly and disabled people, post-acute patients in need of rehabilitation and long-term care, and patients in hospices.

## Background

### Introduction

Since 1980, in almost every OECD country there has been a trend in the reduction of the use of hospitalization [1], as shown by a number of indicators including the number of inpatient days per capita and the number of hospital admissions per resident [2,3]. Where it is possible, out-of-hospital care is widely considered to be less expensive and to fit the needs of patients rather better [4]. Several studies also suggest that policy makers should invest in primary care; these studies show that countries with strong primary care have lower overall healthcare costs, better health outcomes, and a more equitable distribution of resources [5,6].

In OECD countries, this reduction in hospitalization has led to significant resources being directed towards Primary and Community Care (PCC) settings, which now form a fundamental part of most healthcare systems [7,8] and continue to grow in size and importance compared to hospital care. However, in comparison with data on hospital-based healthcare services, data on costs and volumes of PCC services provided to the population are difficult to find and of poor quality. Without such data, it is difficult to describe the evolution of healthcare systems and to assess the impact of different mixes of PCC services in terms of their costs and in terms of the reduction of inappropriate access to hospitals [9]. Moreover, since the network of activities required to provide PCC services usually lacks the physical proximity and common hierarchy that characterizes inpatient services, there is a pressing need to establish data and information systems in order to coordinate and control the delivery of PCC services [10].

Primary and Community Care encompasses a network of various and highly diversified services provided in non-hospital and community-oriented settings. The measurement of quantity and cost for PCC services is complex; there are no widely adopted systems for classifying services such as the system of Diagnosis Related Groups (DRG) used in hospital care. Moreover, PCC services are often physically fragmented in a de-integrated network of different services provided by several actors in different locations [11].

The measurement of PCC services is therefore more difficult than the measurement of services delivered in hospital settings. We herein describe a measurement method for PCC services developed in partnership with PCC professionals that classifies services into 15 types covering the entire range of out-of-hospital care provided in Italy. We then report the data for 2007 and the trends in the four previous years for a sample of 13 Italian Local Health Authorities (LHAs).

The conceptual model of measurement described herein was developed by the authors for a collaborative research study promoted by the *Federazione Italiana Aziende Sanitarie ed Ospedaliere* [Italian Federation of Health Authorities and Hospitals] (FIASO). The discussions with the participating LHAs on the choice of indicators and on the quality of data collected were a central feature of the model.

### The measurement of PCC activity and expenditure

Although several studies have broken down the healthcare expenditure of different countries into its main components, few have analysed the impacts of individual PCC services on overall healthcare expenditure.

Anderson et al. used OECD Health Data from 2005 [12,13] to compare spending on healthcare in OECD countries. They broke down the expenditure into four categories, namely outpatient, inpatient, pharmaceuticals and other medical goods, and other health expenditure [14]. They showed that on average inpatient expenditure accounts for only 40% of total healthcare expenditure, while 45% is accounted for by services for outpatients (30%) and healthcare services for the community (15%), and the remainder by drugs and medical goods (15%).

Orosz and Morgan [15] focused their analysis on the composition of healthcare expenditure for the year 2005 in a sample of 13 OECD countries. They broke the expenditure down by mode of production and found that personal medical services accounted for 70% of overall expenditure, of which 55% was for inpatient care, 15% was for outpatient curative and rehabilitative care, 18% was spent on pharmaceuticals and medical goods, and the remaining 12% was allocated to prevention, public health and community services.

Moreover, international institutions such as OECD and WHO defined a list of indicators and annually collected data to compare health expenditure in different countries. OECD collected and analyzed data for total expenditure on health, prevention and public health, expenditure on inpatient care, expenditure on out-patient care, expenditure on home care, pharmaceuticals and other medical non-durables, therapeutic appliances and other medical durables, current health expenditure by provider, along with data on health expenditure by financing agent/scheme. In the 34 OECD countries (2010 data), expenditure on inpatient care represented 53% of overall health expenditure; 16% was spent on outpatient care, 13% on pharmaceuticals and medical non-durables, 7% on medical non durables and 11% on public health and prevention [16].

The WHO presented instead data on government, private, external, social security and out-of-pocket expenditures on health for 193 countries. Data on health financing were generated from national health accounts that collect expenditure information within an internationally recognized framework. In the average of all WHO countries (2010 data), 54% of health expenditure was financed by governmental expenditure, whereas 46% of expenditure was privately funded. Out of pocket expenditure represented in average 11% of overall health expenditure [17].

Other studies were undertaken to analyse the components of spending on healthcare in individual countries. In the United States, researchers at the Centre for Medicare and Medicaid Services (CMS) analysed national healthcare expenditure accounts, with the goal of measuring the total amount spent on the purchase of healthcare goods and services, by type of service delivered (hospital care, physician services, nursing home care, etc.). In 2007, hospital care represented 37% of total personal healthcare spending in the United States, outpatient services represented 40%, drugs and non-durable medical products 14%, and services for the community 9% [18].

Martin et al. [19,20] analysed data across 23 ‘Programmes of Care’ collected for the years 2003–2005 by the Primary Care Trusts in the NHS in England. Among the largest components of these programmes were primary care, which represented almost 11% of total spending, mental health (12%), and cardiology and circulation problems (10%). Among the other categories, maternity/ gynaecological services received 5% of the budget, gastro-intestinal care received 6%, care for oncological patients 6%, care for diabetic patients 3%, healthy individuals and social care 4%, and dental care 1%.

In general, although these studies used different methods of elaboration (function or type of service delivered, source of funding, or programme of care), they were all based on data that were either published officially or collected previously. The researchers were not able to break the expenditure down further into individual services, nor could they analyse the impact of any allocation of resources on clinical outcomes or other performance indicators.

### Research goals and contributions

This study has two main goals. First, it aims to define a method of data collection and analysis of PCC healthcare services that can be used to describe the consumption of these services by residents in terms of quantity and cost. It is envisaged that the method developed here for the Italian system could also be used in other national healthcare systems. Second, it aims to report detailed data on expenditure and the main indicators of activity for PCC services in the Italian NHS, in order to compare the strategies of different LHAs within the same national context.

We collected primary data directly from LHAs, instead of using previously published or collected secondary data. In so doing, we obtained a level of detail unavailable to previous studies with respect to out-of-hospital care. WHO data focused on the actors financing healthcare expenditure (government, private, social security, out of pocket expenditure), whereas OECD mainly focused on the type of medical good (pharmaceuticals, medical durables and non durables). Neither WHO nor OECD data, however, provided detailed data for typology of service [16,17]. In our study, we broke down overall health expenditure for specific type of PCC service provided to LHAs’ inhabitants.

Moreover, our methodology involved face-to-face interactions among LHAs’ representatives, which permitted a sense-making process to take place that was useful for the proper definition of indicators, to assess the reliability of the data, and to provide benchmarking information for the LHAs.

In summary, the process of primary data collection of both expenditure and activity indicators outlined in this paper and the emphasis on assessing data reliability by means of continuous interactions among researchers and LHAs’ professionals make our methodology applicable to all Italian Regions and to OECD countries.

## Methods

### The collaborative research procedure

The study was conducted by collecting quantitative data on the costs and activities of 13 Italian LHAs participating in a collaborative research study on the management of PCC services, organized by the FIASO. The FIASO is the main association of public sector healthcare organizations and has 160 member organizations (either LHAs or public Independent Hospitals [IHs]) out of the 249 public healthcare organizations in Italy. A brief overview of the main characteristics and recent reforms of the Italian NHS is provided in Table 1.

**Table 1:** The Italian NHS: key features and recent reforms

**Foundations**	The Italian National Health Service (INHS) was established in 1978 and modelled after the British NHS [21,22]. Coverage is universal and theoretically uniform throughout the country [21], and both its financing and delivery are mostly public.
**Tiers**	The INHS has three tiers: Central Government, responsible for guaranteeing essential levels of assistance for every citizen; 21 Regional Governments; and 154 Local Health Authorities (LHAs and 95 Independent SSN Hospitals (or IHs; similar to British NHS Trusts). The LHAs are regional public agencies that manage healthcare services for subsets of the regional population in a defined geographical area. Each LHA serves an average population of about 390,000 inhabitants and manages an average budget of 662 million euros, partly for in-house provision, partly to purchase services from public IHs (on average, 132 million euros per LHA, [23]) and from private contracted providers (on average, 128 million euros per LHA, [23,24]).
**Reforms**	Over the last 15 years the INHS has undergone a series of reforms that have introduced quasi-markets, regionalization, and managerialism. A quasi-market system implies that money follows the patient: LHAs pay a provider for their resident’s consumption of healthcare if this is not provided directly by themselves. Patients are free to choose other public or private providers from elsewhere in the country and services are paid for by their LHA [25]. The LHAs are usually funded on a capitation basis and each LHA is expected to reimburse other LHAs, IHs, and accredited private providers for services supplied to its residents [25]. However, regionalization reforms have led to significant variability in how this model is implemented, mainly in the number of facilities directly managed by LHAs, in the degree of autonomy of LHAs in strategic and operational decisions, and in the modification of the capitation funding scheme to match historic expenses.

**Figure 1:**
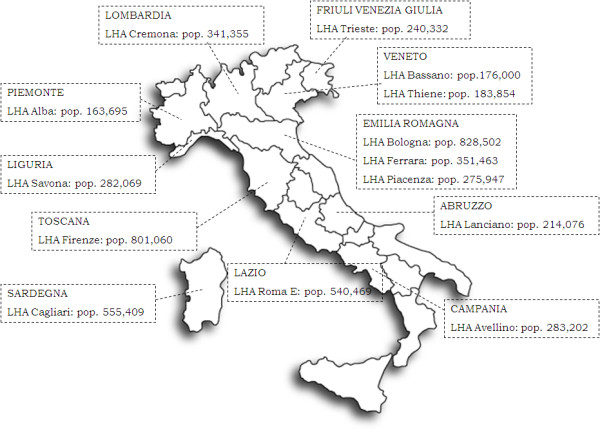
The population and geographical location of the 13 LHAs.

The population covered by the 13 LHAs participating in the research was 5.1 million, which represented 9% of the total population of Italy. The combined yearly budget of the 13 LHAs is equivalent to 8.7 billion euros, almost 9% of the Italian NHS expenditure. The LHAs participating in the study represent 10 of the 20 Italian regions and cover the length and breadth of the country; as in northern regions information systems aimed at collecting and analyzing data are in general more sophisticated than in the southern regions, the sample is opportunistic and some regions (Veneto and Emilia-Romagna) are over-represented, while others in the South are not represented. An overview of the populations and locations of the 13 LHAs is provided in Figure 1.

During the preliminary collaborative work, both the authors and some of the general managers of the member LHAs identified the two main goals described above, of collecting data about the healthcare services consumed by residents, and of providing a forum in which to discuss benchmarking and the differences between LHAs.

Each of the participating LHAs designated three managers to be representatives in the collaborative research; a total of 39 managers were involved in the research activity, usually the financial controller, the head of the primary care department, and a representative of the top management. The authors of the present paper were nominated by the FIASO coordinators of the research programme and undertook the role of managing the collection and analysis of the data and of facilitating the collaborative research process. All LHAs gave their approval to the research.

First, we drafted an initial list of indicators that was discussed in the first focus group meeting with representatives of the LHAs. Subsequently, all 39 participants met the authors for four two-day workshops of eight hours per day. These workshops had several aims. The first workshop was used to discuss the draft list of indicators and to define a manual including a short description of each indicator and the rules of data collection. The second and third workshops were used to discuss and verify the reliability of the data collected. The LHA managers discussed the inter-LHA inconsistencies, which could have been caused either by data collection from different sources, or by the misalignment between the definitions of those services provided by the LHAs. Given the complexity of the PCC services and the autonomy enjoyed by the LHAs, indeed, the possibility existed that PCC services that had identical denominations were often not related to the same typology, quantity, and quality of the services offered to citizens [26].

One of the most time-consuming tasks that took place during the workshops was the reduction in the misalignment of the definitions and the contexts of each PCC service in each LHA; it was commonly the case that the representatives of each LHA labelled the same service using different names, and in some cases labelled services that were very different with the same name. This was more evident for those services that were characterized by a lack of national standards, and for which LHAs were allowed a degree of autonomy (such as mother and child health, home care) than for the traditional services of primary care (general practitioners (GPs), prevention, and public health). The final workshop was used to discuss the resulting evidence and to debate the managerial implications for the LHAs.

The collaborative research took more than a year, from the first workshop in May 2007 to when the final results of this study were presented in July 2008. During this period, all the teams from the 13 LHAs were involved in data collection and ongoing interaction with the authors, working an average of 15 days in total over the period of the study.

The representatives of each LHA were asked to collect data on the use of healthcare services by their resident population. This resulted in a list of 303 indicators on quantity, appropriateness, costs, provision setting (hospital, emergency services, home care, etc.), and the nature of the provision (in-house, public or private external providers). The LHAs are all purchasers or providers of their residents’ healthcare consumption under the Italian NHS, and they therefore have access to administrative data on every type of healthcare service consumed by their resident population and delivered by any type of provider, with the only exception being private out-of-pocket expenditure, which in Italy accounts for 23% of spending in the entire healthcare sector according to OECD data [16].

### The data collection

The method of analysis follows the collection of a set of 303 indicators related to the costs of PCC services and the activities concerned. Cost and activity indicators were computed on the basis of the healthcare services consumed by the residents of the LHAs during the year of the study that were funded by the Italian NHS, and that were provided by any type of provider (directly by the LHA, by IHs, by accredited private providers, or by other LHAs). In some regions LHAs are significantly involved in the provision of healthcare services, whereas in Lombardy Region there is a clear purchaser–provider split, and LHAs only assume the role of purchaser. Despite this difference in the quantity of services provided directly, all the LHAs receive per capita funding by their regional government to provide or to purchase healthcare services for their inhabitants. This guarantees homogeneity when comparing the resources used by the LHAs to finance their healthcare services.

The LHAs extracted data from their different information systems such as management accounting systems and customized in-house systems. One of the requirements of the procedure used for data collection was that the information on aggregate costs drawn from the final balance sheet matched the sum of the costs of the single services obtained from analytical accounting. Where there were differences between the two total costs, the LHAs were asked first to check for possible causes of the mismatch, then to improve the quality of the data used, and then to split the cost differential in each area of activity in proportion to its relative weight in comparison with the total expenditure. Missing data for single indicators related to one or more LHAs are due to the unavailability of data from the respective management information systems.

In order to evaluate the main trends for each indicator, the LHAs were requested to collect data related to two different years, 2007 and 2003, or data related to the closest available year (2004), if data for 2003 were unavailable. By taking this approach, we were able to assess the percentage variation of each indicator within the considered period. To neutralize the effect of inflation on monetary indicators, all the data were scaled to the year 2007 using the appreciation rates provided by the Italian National Statistical Institute [26].

In order to account for differences in the demographic structure of the LHAs, we weighted inhabitants’ use of outpatient visits to specialists, and examinations, pharmaceuticals, and hospital admissions, based on age and sex, adopting the same methodology used by the Italian NHS to weight the per capita funding to regional healthcare services in 2007 [27]. These criteria are provided in Additional file 1: Appendix 2.

## Results

### LHA expenditure on types of PCC service and trends in the period 2003-2007

The LHAs within the sample spent in year 2007 an average of 1,682 euros per inhabitant; this covers any kind of healthcare service offered to residents by any type of public and private provider within the Italian NHS. This value is measured as full costing for each type of service and, therefore, includes expenditure deficits LHAs may create by having costs higher than their funding. On average deficit of the LHAs within our sample was 2.5% of the overall expenditure [28]. Table 2 shows the breakdown of the average cost per inhabitant for the 15 types of service in the 13 LHAs. The description of the content of each type of service is provided in Table 3.

**Table 2:** Health expenditure of the 13 LHAs broken down by type of service (2007 data)

**Type of service**	**Average cost per inhabitant 2007 (in euros)**	**% on total expenditure**	**Standard deviation in euros(and%on average cost per inhabitant**)	**Average yearly percentage variation between 2003 and2007**
Hospital admissions	697	41%	89 (13%)	2%
Pharmaceutical	273	16%	37 (14%)	2%
Outpatient visits and examinations	220	13%	76 (35%)	7%
General Practitioners (including night service)	103	6%	9 (9%)	3%
Admissions of elderly people to residential structures	77	5%	39 (51%)	4%
Mental healthcare	59	4%	22 (37%)	2%
Prevention, public health and screening	56	3%	14 (25%)	3%
Emergency services	49	3%	26 (53%)	6%
Rehabilitation	28	2%	27 (95%)	9%
Disabled people	35	2%	24 (68%)	9%
Prosthesis	27	2%	11 (40%)	4%
Home healthcare	22	1%	11 (50%)	7%
Mother and child	18	1%w	12 (65%)	3%
Dependencies	16	1%	4 (25%)	17%
Hospice	3	0%	3 (118%)	6%
Overall cost per inhabitant	1,682	100%	171 (10%)	3%

**Table 3:** Description of the type of service

**Type of service**	**Description of the service**
Hospital admissions	Costs for ordinary and day hospital admissions. It includes all the costs for inpatients and is measured through DRG system
Pharmaceutical	Costs for drugs distributed directly or through local private chemists
Outpatient visits and examinations	It includes the costs for specialist visits in hospital and ambulatory facilities, diagnostic exams and laboratory exams to outpatients
General Practitioners (including night service)	It includes the costs for General Practitioners, General Practitioners for children under 14 (“Pediatri di Libera Scelta” in the INHS) and 24 hours, night service
Admissions of elderly people to residential structures	Costs for institutionalization of old people (>65) in intermediate facilities and for access to daily structures, including healthcare assistance
Mental healthcare	Costs for institutionalization and ambulatory services for mental care. It does not include neuro-psychiatry (included in mother and child) and dependencies
Prevention, public health and screening	Costs for screening programs (e.g. Colon-rectum cancer and breast cancer) and more general expenditure for illness prevention managed by LHAs
Emergency services	It includes emergency departments and ambulance services
Rehabilitation	Costs for long term care in residential, intermediate structures and in ambulatory facilities. It does not include assistance in hospital departments for inpatients (included in hospital admissions)
Disabled people	Costs for institutionalization of disabled people in residential structures and ambulatory services, including eventual vouchers to be spent by disabled people
Prosthesis	Costs for providing and managing prosthesis
Home healthcare	Mono-professional or multi-professional (eg. GPs, nurses, specialists) home care
Mother and child	Costs for ambulatory services including family planning clinics, neuro-psychiatry for children and community support to lone parents
Dependencies	It includes ambulatory services for dependencies.
Hospice	Costs for admissions in hospice structures
Overall cost per inhabitant	Full costing for citizens’ healthcare. It includes administrative and general costs (eg. Costs for LHAs’ top managers), which are shared in percentage to all the other services. It also includes eventual health expenditure deficits (which have an average value within our sample of 2.5% of the overall expenditure) [28].

The costs for inpatients, i.e., for both hospital admissions and emergency services, represented 44% of the overall costs, with an average expenditure within the sample of 746 euros per inhabitant (697 euros for hospital admissions and 49 euros for emergency services). The costs for outpatient services and PCC accounted for the remaining 56% of the total budget, with an average expenditure of 936 euros per inhabitant. Specifically, the expenditure on outpatients’ drugs and medical goods (including prostheses) represented 16% of the total costs, with the expenditure on outpatients’ visits to specialists and on diagnostic examinations being 13%, primary care (including prevention and public health) 9%, and intermediate structures (for rehabilitation, elderly people, disabled people, mental health) 11%.

The LHAs’ expenditure is strongly focused on a few types of service: the top five types of service in order of decreasing expenditure per inhabitant (hospital admissions, pharmaceuticals, visits to specialists and for examinations, GPs, admissions of elderly people to residential structures) represented more than 81% of the total expenditure.

The comparison between expenditure data of Italian LHAs and the average of OECD data [16] shows that the Italian NHS designed an important trajectory of investment in Primary and Community Care services, along with a robust shift of resources from hospital to outpatient care. Expenditure in inpatients, hospital services (including also emergency services) represented in the average of our sample of Italian LHAs only 44% of the overall expenditure, versus an average value within the OECD countries of 53% of overall healthcare expenditure. Looking at specific components of the aggregate value, expenditure on pharmaceuticals was higher in the Italian sample than in the average of OECD countries (16% vs. 13% of overall expenditure), whereas Italian LHAs invested a limited amount of resources in Prevention and Public Health (only 3%).

The average annual costs increased over the period of analysis (2003–2007) by 3% (Table 2). This headline rate varied significantly according to the particular type of service: among the top five activity ambits, the costs of hospital care increased by 2% per year over the period of analysis, whereas the costs of outpatient visits to specialists and for examinations had the highest annual growth rate, of 7%. These results suggest that in future an ongoing reduction in hospital admissions and an increase in outpatient and PCC consumption may be expected. In the other areas of care, the expenditure on services related to dependency exhibited the highest growth rate at 17%. The expenditure on services for rehabilitation and for disabled people also increased significantly, with a growth rate of 9%.

The yearly variation in expenditure between 2003 and 2007 was related to a parallel variation in LHAs’ indicators of activity concerning both hospital and primary and community care (Table 4). The overall number of hospital admissions yearly decreased by 3% in the period 2003–2007. This reduction in the volume of hospital admissions was also related to higher appropriateness in the access to hospital services: inappropriate access to emergency services decreased in average by 3% and inappropriate hospital admissions decreased by 4% within the sample. On the contrary, activity indicators related to primary and community care services showed important annual growth rates in the period 2003–2007. The access to mental health services, for example, increased by 8%, the access to mother and child structures by 6%, and the number of home visits by 9%.

**Table 4:** Average activity indicators for hospital and primary and community care (2007 data and yearly variation in the period 2003–2007)

**Activity indicator**	**Average value 2007**	**Standard deviation and%on the average value**	**Average yearly percentage variation between 2003 and2007**
Hospitalization rate per 1,000 inhabitants	194	42 (22%)	−3%
Inappropriate access to Emergency Services per 1,000 inhabitants	98	68 (70%)	−2%
Inappropriate hospital admissions (51 DRGs) per 1,000 inhabitants	20	13 (67%)	−4%
Access to mother and child structures per 1,000 inhabitants	174	128 (74%)	+ 6%
Access to mental health structures per 1,000 inhabitants	195	171 (77%)	+ 8%
Home care visits per 1,000 inhabitants	425	161(38%)	+ 9%

### Variance in expenditure and activity among LHAs

The LHAs within the sample exhibit considerable differences both in terms of their overall health expenditure and in their volume of activity. The variance in expenditure within the sample was measured in terms of the standard deviation and is shown in Table 2. Overall, the expenditure on healthcare per inhabitant in 2007 varied within the sample from 1,407 to 1,989 euros, with a standard deviation of 171 euros. The services with the highest standard deviation were hospital admissions (89 euros), and outpatients’ visits to specialists and examinations (76 euros), whereas the area with the lowest standard deviation (among the most relevant) was GPs (9 euros). Non reported analysis parcelling out administrative costs from each type of service shows a very high standard deviation of administrative costs too.

Next, the variation in the LHAs’ activities was assessed using several indicators related to the LHAs’ hospital and PCC activities. Average findings are presented in Table 4, whereas Table 5 provides a more detailed analysis of variance within each of the 13 LHAs; it can be seen that there are considerable differences among the sample. In Table 6 a description of the methods used to measure each indicator is provided.

**Table 5:** Indicators of hospital and PCC services within each LHA of the sample (2007 data)

**Indicator**	**LHA 1**	**LHA 2**	**LHA 3**	**LHA 4**	**LHA 5**	**LHA 6**	**LHA 7**	**LHA 8**	**LHA 9**	**LHA 10**	**LHA 11**	**LHA 12**	**LHA 13**	**Average value**
Hospitalization rate per 1,000 inhabitants	188		173	142	150	222	284	186	191		225	191	185	194
Inappropriate access to Emergency Services per 1,000 inhabitants	136	31		137	57		60	165	41	35	100	228		99
Inappropriate hospital admissions (51 DRGs) per 1,000 inhabitants	11	22	14	15	17	40	22	5	38	10		20	33	21
Access to mother and child structures per 1,000 inhabitants	432	53	30	222	175	111	253	37	78	104	341	250		174
Access to mental health structures per 1,000 inhabitants	466	275	237	84	101	42	244	43	311		192	380	28	200
Home care visits per 1,000 inhabitants	514	594	87	444	477	221	530	393	579	494	231	531		425

**Table 6:** Description of the indicators

**Indicator**	**Description**
Hospitalization rate per 1,000 inhabitants	Number of admissions to public and private hospitals per 1,000 LHA inhabitants in 2007. Data have been weighted for inhabitants’ age and sex. They have been also validated by the Regions.
Inappropriate access to Emergency Services per 1,000 inhabitants	White code access to Emergency Services per 1,000 LHA inhabitants in 2007. Data have been weighted for inhabitants’ age and sex.
Inappropriate hospital admissions (51 DRGs) per 1,000 inhabitants	Overall number of admissions to public and private hospitals for a list of 51 Diagnosis Related Groups listed by the Italian Ministry of Health as at high risk of inappropriateness, for 1,000 LHA inhabitants in 2007. Data have been weighted for inhabitants’ age and sex.
Access to mother and child structures per 1,000 inhabitants	Overall number of registered accesses to clinics or other structures devoted to mother and child health managed by LHAs for 1,000 non-weighted LHA inhabitants in 2007.
Access to mental health structures per 1,000 inhabitants	Overall number of registered accesses to mental health structures managed by LHAs for 1,000 non-weighted LHA inhabitants in 2007.
Home care visits per 1,000 inhabitants	Overall number of home visits provided by LHA PCC services or by private providers financed by LHA for 1,000 non-weighted LHA inhabitants in 2007.

With respect to hospital care, hospitalization rates varied from 142 to 284 admissions per 1,000 inhabitants per year, against a national standard recommended by the Ministry of Health of 180 admissions per 1,000 inhabitants; in average, a resident of the LHA with the highest hospitalization rate is exactly twice as likely to stay in a hospital than a resident of the LHA with the lowest hospitalization rate.

This variability increased while taking into account indicators of appropriateness, in terms of admissions to hospital and emergency services. The number of inappropriate hospital admissions varied within the sample from 5–40 admissions per 1,000 inhabitants, with a standard deviation equal to 67% of the average value of the sample. Rates of inappropriate admissions were calculated for 51 DRGs which are considered by the Ministry of Health to be inappropriate when requiring hospital admission. For example, in the case of cataract surgery, according to the Italian Ministry of Health, a patient should normally be treated in day surgery in hospital or as an outpatient, rather than as an inpatient.

A further indicator of appropriateness in the provision of healthcare services is the number of white codes in emergency departments, which are defined as accesses to emergency services which could be more appropriately treated in non-emergency settings (e.g., General Practitioners’ ambulatories or continuity of care services). This indicator varied from 31–228 per 1,000 inhabitants in the LHAs of our sample, with a standard deviation equal to 70% of the average value.

Variation was also higher for indicators of primary and community care services. The average number of accesses to mother and child services varied within the sample from 30 to 432 accesses per 1,000 inhabitants, with a standard deviation equal to 74% of the average value. The number of accesses to mental health services ranged from 28 to 466 within the 13 LHAs, with a standard deviation value of 77% of the average value.

Moreover, variance within the sample was assessed by breaking down PCC expenditure of each LHA into three main groups of services: primary and ambulatory services, intermediate structures involving patient admission and two focused and specialized departments, namely Prevention and Public Health and Mental Healthcare, that are organizationally homogeneous in all the LHAs. Primary and ambulatory services consist of activities or processes that attempt to ensure primary assistance and continuity of care for patients without requiring any institutionalization. They include GPs, paediatricians, home healthcare and nursing home care, ambulatory mother and child services. Intermediate services, instead, are based on the physical presence of residential facilities and include services that aim to assist patients after their discharge from hospital, either in residential facilities for rehabilitation and long-term care or in ambulatory settings, such as structures for elderly people, disabled people, for rehabilitation and long-term care, hospice care. We decided then to keep distinct ‘Prevention and Public Health’ and ‘Mental Healthcare’ from the other types of PCC services, as they represent in the Italian context highly institutionalized departments that are traditionally homogeneous within the national sample of Italian LHAs, due to normative pressures separating the boundaries of those organizational departments from other PCC services. Including those departments in the other aggregations of PCC services could therefore risk to bias the comparison among LHAs in our sample.

**Figure 2:**
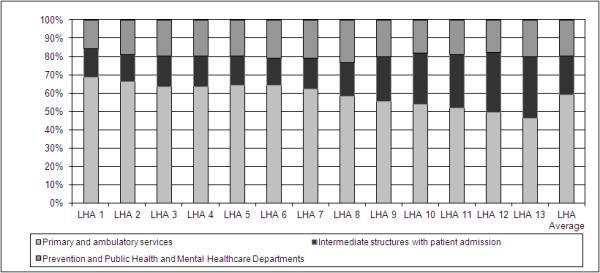
Composition of PCC expenditure within the sample (2007 data).

The data reported in the graph (Figure 2) show that the costs of primary and ambulatory services vary from 46–69% of total PCC expenditure, the costs of intermediate structures vary from 15–34%, while the costs of departments of Prevention and Public Health and Mental Healthcare are substantially homogeneous in all the LHAs (from 17–22%). This evidence suggests the presence of a high degree of variance in the strategies adopted by LHAs in defining the mix of PCC services they provide to their inhabitants.

In addition, Table 7 reports aggregate analysis of the yearly average variation for the expenditure on groups of PCC services within the period 2003–2007. Expenditure for primary and ambulatory services increased in average by 4%; looking at the single components of expenditure, resources for home care increased more than resources for primary care services (7% of yearly growth versus 3%). Expenditure for intermediate services showed an average increase of 5%, driven in particular by the high growth rate of resources devoted to rehabilitation structures (9%). Prevention and Public Health and Mental Health Departments, instead, only slightly increased their annual level of expenditure in the period 2003–2007 (2%).

**Table 7:** Variation in the composition of PCC expenditure in the period 2003–2007

**Aggregation of services**	**Yearly percentage variation in expenditure 2003-2007**
Primary and ambulatory services	+ 4%
of which: primary care	+ 3%
of which: home care	+ 7%
Intermediate structures with patients’ admission	+ 5%
of which: structures for old people	+ 4%
of which: rehabilitation structures	+ 9%
Prevention and Public Health and Mental Health Departments	+ 2%
of which: mental healthcare	+ 2%
of which: prevention and public health	+ 3%

The list of the indicators collected by the 13 LHAs during the collaborative research, together with their median and average values, standard deviations, and yearly growth rates, are given in Additional file 1: Appendix 1.

## Discussion

### LHA expenditure on types of PCC service and trends in the period 2003-2007

Through a detailed analysis of healthcare consumption, which looked both at costs and at services provided by a sample of 13 Italian LHAs, we collected data on health expenditure and outputs related to PCC services. Our results show that the financial resources devoted to PCC are greater than those devoted to hospital inpatient and emergency services, representing on average 56% of the total expenditure of LHAs on their inhabitants’ healthcare consumption. Although the expenditure on PCC services exceeds that on hospital care in our sample, the mix of services is highly heterogeneous among the LHAs. Most of the LHAs’ expenditure is focused on a few services (hospital admissions, pharmaceuticals and outpatients’ services), whereas a number of other services with increasing health and social relevance (e.g., services for elderly people, disabled people, mother and child, and hospice care) receive comparatively few resources. Our findings show that LHAs tend to focus their activity on traditional types of PCC services (e.g., GPs, outpatients’ visits to specialists and diagnostic examinations) and allocate only marginal resources to those services that provide care for a growing range of citizens’ needs, from disability to hospice care.

Our findings on the annual trends in healthcare expenditure show that there is strong growth in the percentage of outpatient visits to specialists and for diagnostic examinations; this category represents the third service in terms of overall costs (220 euros per inhabitant) and shows a 6% growth rate, which is much higher than those of the other main PCC services. The weakness of many LHAs in managing the level of consumption in this category and integrating it with other services (such as services provided by GP practices and visits to specialists and examinations in hospital) appears to be contributing to the increase in this expenditure, with the risk of clinical path fragmentation, the duplication of costs, and high levels of inappropriate visits [28].

### Variance in expenditure and activity among LHAs

There is a high degree of variance in the overall expenditure of LHAs and in their mix of PCC services. Moreover, there is a different mix of expenditure on tiers of care across the LHAs. Specifically, two distinct strategic orientations have emerged with regard to the adoption of PCC services by LHAs: one group of LHAs mainly invested in ambulatory and home-based primary care services (GP networks, community-based services, home and ambulatory care) in order to increase the number of low-complexity settings (with GPs and nurses as case managers), to integrate health and social assistance for chronic patients, and to reduce the inappropriate use of hospital admissions and emergency services. A second group of LHAs prioritized the allocation of resources to focus on intermediate inpatient structures for specific types of patients, namely elderly and disabled people, post-acute patients needing rehabilitation and long-term care, and those in hospices. In this case, the LHAs tended to increase the degree of appropriateness of inpatients with admissions using nursing-based structures.

Evidence of the two strategic orientations within the Italian context arises from the analysis of both Regional and LHAs’ strategies. At the Regional level, for example, two of the most populous and wealthy Regions in northern Italy chose different strategic orientations for developing their PCC services. Lombardia Region mainly focused on intermediate, residential structures for older and disabled people, whereas the contiguous Emilia-Romagna Region mainly invested in ambulatory and home-based primary care services. Some few indicators clearly show this difference in strategic orientation. In Lombardia Region there is an endowment of almost 6.5 beds in structures for older people per 1,000 inhabitants, whereas in Emilia-Romagna there are almost 3.2 beds per 1,000 inhabitants. On the contrary, Emilia-Romagna outshines Lombardia for accesses to home care services, with an average value of 454 versus 368 home care visits per 1,000 inhabitants [29].

Those strategic orientations are also evident in the LHAs of our sample. LHA1 is a LHA in Emilia-Romagna Region, with a strong focus on primary and ambulatory services (69% of overall PCC expenditure versus an average of 59% within the sample). Looking at activity indicators, LHA 1 presents a high degree of access to home care services (514 versus an average of 424) and to mother and child services (432 versus an average of 160). Those indicators are related to a limited endowment of intermediate structures for older people (3,241 days in residential structures per 1,000 inhabitants > 65 years versus an average of 5,423 within the sample) and for disabled people (78 days in residential structures for disabled per 1,000 inhabitants versus an average of 115). On the contrary, LHA 11 is a LHA in Lombardia Region and has a strong focus on expenditure in intermediate care structures (28% of overall PCC expenditure versus an average of 21% within the sample). Services for primary and ambulatory services are limited, as it is suggested by the low degree of access to home care services (231 accesses per 1,000 inhabitants versus an average of 424). LHA 11, however, widely invested in intermediate structures for older people (8,652 days in residential structures per 1,000 inhabitants > 65 years versus an average of 5,423) and for disabled people (128 days in residential structures for disabled per 1,000 inhabitants versus an average of 115).

Both strategies have an impact on the local hospital network concerned. In the first case (focus on home and ambulatory care), there is still a need for some local community hospital, in order to provide timely care to patients with acute needs. In the second case (focus on intermediate structures), the opportunity exists for an additional reduction in the local hospital network. Intermediate structures are endowed with medical and nursing personnel and can often also provide appropriate care for some of the chronic and elderly patients’ acute phases, thus a further reduction in inpatient hospital assistance is possible [11]. This second scenario is less problematic in terms of its implementation, but tends to require a greater number of separate PCC services, which necessitates a stronger strategic commitment from the LHA.

Moreover, the data show that the LHAs in the sample are homogeneously distributed along the entire range of potential PCC strategies, ranging from a focus at one end of the spectrum on home and ambulatory care to a focus at the other end on the use of intermediate structures. During our discussions with LHAs managers, it appeared that these opposing strategies had emerged over time, rather than having been consciously and explicitly planned. For instance, one representative stated: “We have never thought about PCC in this way, we were only concentrating on decreasing the hospital market share and on developing community and primary care”.

However, the choices made by the LHAs are often driven by path dependency on previous structural or professional resources rather than by their definition of strategic priorities. Those LHAs that are endowed with former hospital or ambulatory structures tend to have a large number of intermediate care structures. Those LHAs that do not have these structural facilities are instead more suited to investing in ambulatory and home care and to developing GP structures. On the other hand, community-based specialist departments and medical centres, such as departments of Prevention and Public Health and Mental Healthcare, which in Italy are highly regulated by controls that are institutionalized centrally, register much less variability. As shown by our data, they tend to maintain the historical consumption of resources devoted to respect the planned staff, equipment and facilities standards, with limited variation among LHAs.

Path dependency is often related to a weak organizational consciousness and a lack of explicit service portfolio policies [30]. In the Italian NHS, this issue is part of a generally poor debate about how to organize PCC services and how to allocate their resources, which is in contrast to the richness of the literature and the quality of the regulation related to hospital standards.

## Conclusions

The model of this collaborative research study and the results from the collected data suggest that there are strategic implications for both LHAs and the Italian NHS as a whole, from which many other healthcare systems may draw some lessons. There has been an increasing shift of services and resources from inpatient hospital settings to settings based on outpatient community and primary care. Many LHAs are introducing innovative PCC services and have the scope to develop these further. In this process of transformation, LHAs have the flexibility to identify a suitable mix of PCC services and settings on which to focus their financial and organizational resources. In a context dominated by financial constraints, both the service portfolio and the organizational evolution of PCC services must tackle the emerging needs of elderly and chronic patients and the increasing expectations of the population as a whole [22]. Our findings suggest the presence of different balances between primary and intermediate care. Some LHAs prioritize primary care and focus on PCC services (home care, community care, and ambulatory care): in these LHAs, a central role is played by GPs coordinated in group practices, and there is a progressive involvement of nurses acting in the role of case managers for chronic patients. Other LHAs prefer to focus their resources on intermediate structures, each providing care for specific clusters of patients (e.g., elderly people, post-acute and chronic patients, disabled people) and mirroring hospital settings through the establishment of a network of non-hospital structures with a higher level of appropriateness in relation to their inhabitants’ needs.

However, whether and to what extent these different balances are driven by the LHAs’ strategic choices is unclear. Moreover, these different balances may be the result of emergent, unplanned strategies that depend on the LHA’s path dependency on previous structural or organizational resources. As suggested by literature on management, path-dependent resources (e.g., structures, specialist technical expertise, and unique capabilities) are rooted in the history and culture of organizations, and there is a high likelihood that they will be perpetuated within those organizations [31-33].

We have herein proposed an innovative methodology of data collection for PCC services, which was developed as part of a FIASO collaborative research study, through the analysis of data on health consumption and costs within 13 Italian LHAs. The participants and authors identified a list of more than 300 indicators of PCC services within the Italian NHS. The aim was to tackle the complexity of understanding the consumption of PCC services through an intense interaction with the owners of the data. Whereas previous studies compared and elaborated aggregated and analytical data collected by separate and independent sources [13,14], in this collaborative research study the authors acted as facilitators and coordinators and interacted with 39 representatives from the participating Italian LHAs for almost a year.

This measurement approach can be applied to LHAs whose management information systems are at different stages of maturity. For example, the costs and activities of LHAs with good management information systems can be compared at an analytical level by using more than 300 indicators; however, it is possible for any LHA to provide a synthetic representation of its healthcare consumption and of key activity indicators by following our approach. Moreover, for the representatives of the LHAs involved, the process of data collection was an important opportunity to identify and compare costs, outputs, and indicators of the quality of the healthcare services they provide to their inhabitants, and to make sense of the data by discussing and interacting with other LHA representatives and the authors.

As shown by the results reported herein, after more than 15 years of progressive resource shifting from hospital to PCC settings [14,15], the resources devoted to the full gamut of out-of-hospital care are greater than those allocated to traditional hospital services (56% vs. 44%), but the mix of services provided by the LHAs appears to be highly heterogeneous. The measurement of PCC is important because PCC includes areas of healthcare that are increasing in terms of expenditure and impact on the health of citizens, but these are often inadequately measured and evaluated by LHAs [28]. The management of LHAs should collect and analyse data about their inhabitants’ consumption and the PCC that they provide, in order to ascertain the mix of services most suitable for their population’s health needs. Managers who know which services healthcare organizations are providing, and at what cost, will be better able to satisfy their population’s needs, to assess the impact of these services on health outcomes, and to manage the complex network of providers of all these services. We suggest that future research should investigate whether and to what extent primary and ambulatory services and intermediate structures can be considered as substitutable or whether these services have different impacts in terms of the population’s health outcomes and quality of care.

In conclusion, the data reported in this study, the discussion, and concluding suggestions indicate that LHAs’ strategies regarding the provision of PCC will play a vital role in the future direction of healthcare organizations and health systems. From this perspective, PCC cannot be considered to be a single service, but to be rather a combination of several services that contribute in variety of ways to the achievement of multiple healthcare goals. We hope that the model proposed in this article can be used as a first step in analysing wider and different contexts, with the aim of ascertaining which combination of PCC services enables LHAs to obtain the best results in terms of effectiveness of care and ability to satisfy the population’s diverse healthcare needs.

## Abbreviations

LHA: Local Health Authority; INHS: Italian National Health Service; PCC: Primary and Community based-care services.

## Competing interests

This FIASO collaborative research is supported by an unrestricted grant from *Roche S*.*PA*. *Italia*.

## Authors’ contribution

Authors' names are listed in alphabetical order. All authors equally contributed to the paper. All authors read and approved the final manuscript.

## Authors’ information

Francesco Longo is Associate Professor at the Department of Institutional Analysis and Public Management of Bocconi University, Milano

Domenico Salvatore is Assistant Professor at the Parthenope University, and researcher at Istituto SDN Napoli.

Stefano Tasselli is PhD candidate at the Judge Business School of the University of Cambridge and Research Fellow at Cergas-Bocconi.

## Pre-publication history

The pre-publication history for this paper can be accessed here:

http://www.biomedcentral.com/1472-6963/12/393/prepub

## Supplementary Material

Additional file 1 **Appendix 1 the analytical data of costs and activities collected within the 13 LHAs (2007 data).** Appendix 2 the criteria used to weight the inhabitants’ consumption of outpatient visits to specialists and examinations, pharmaceuticals, and hospital admissions, based on age and sex. Click here for file
