# Supplementary material for: The growth and composition of primary and community-based care services. Metrics and evidence from the Italian National Health Service
Source: BMC Health Serv Res. 2012 Nov 13;12:393. doi: 10.1186/1472-6963-12-393 (PMC3508936; doi:10.1186/1472-6963-12-393)
Supplement: Additional file 1 — Appendix 1 the analytical data of costs and activities collected within the 13 LHAs (2007 data). Appendix 2 the criteria used to weight the inhabitants’ consumption of outpatient visits to specialists and examinations, pharmaceuticals, and hospital admissions, based on age and sex. [file 1472-6963-12-393-S1.doc]

**Appendix 1: Analytical data of costs and ACTIVITIES COLLECTED WITHIN 13 LHA (2007 data)**

***Indicators related to demographic structure of population and key indicators of costs and activity***

| **Indicator** | **Code** | **Median** | **Average** | **Standard deviation** | **Number of answers** | **Median annual variation** | **Average annual variation** | **Standard deviation** | **Number of answers** |
| --- | --- | --- | --- | --- | --- | --- | --- | --- | --- |
| Number of inhabitants | a1 | 283.202 | 389.033 | 222.622 | 13 | 1% | 1% | 2% | 13 |
| Inhabitants years 0-1 | a2 | 4.298 | 3.994 | 1.840 | 13 | 2% | 2% | 1% | 13 |
| Inhabitants years 1-4 | a3 | 9.704 | 12.577 | 8.285 | 13 | 1% | 2% | 1% | 13 |
| Inhabitants years 5- 7 | a4 | 8.267 | 9.890 | 5.183 | 13 | 2% | 2% | 2% | 13 |
| Inhabitants years 8-14 | a5 | 16.015 | 21.822 | 12.024 | 13 | 1% | 1% | 1% | 13 |
| Inhabitants years 15-24 | a6 | 25.339 | 33.726 | 17.886 | 13 | 0% | 0% | 1% | 13 |
| Inhabitants years 25-44 | a7 | 88.945 | 117.345 | 68.725 | 13 | 0% | 0% | 2% | 13 |
| Inhabitants years 45-64 | a8 | 75.886 | 102.706 | 59.997 | 13 | 0% | 1% | 2% | 13 |
| Inhabitants years 65-74 | a9 | 39.658 | 45.056 | 26.261 | 13 | 1% | 2% | 2% | 13 |
| Inhabitants years 75-89 | a10 | 32.431 | 37.981 | 24.363 | 13 | 2% | 2% | 3% | 13 |
| Inhabitants over 90 years | a12 | 3.696 | 4.202 | 3.109 | 13 | 3% | 3% | 4% | 13 |

***Overall expenditure for healthcare services consumed by LHA inhabitants***

| **Indicator** | **Code** | **Median** | **Average** | **Standard deviation** | **Number of answers** | **Median annual variation** | **Average annual variation** | **Standard deviation** | **Number of answers** |
| --- | --- | --- | --- | --- | --- | --- | --- | --- | --- |
| Total expenditure for services directly produced by LHA per inhabitant (A) | a15 | € 1.513 | € 1.524 | € 166 | 13 | 3% | 2% | 2% | 13 |
| Patients’ mobility, as the expenditure for services consumed by inhabitants outside LHA boundaries or in public and private structures not directly managed by LHA (B) | a101 | € 266 | € 299 | € 141 | 13 | 1% | 2% | 5% | 13 |
| Expenditure for services consumed by other LHA inhabitants in LHA structures (C) | a102 | € 93 | € 123 | € 88 | 13 | 1% | 0% | 5% | 13 |
| Expenditure for healthcare services consumed by inhabitants not considered in previous categories (D) | a103 | € - | € 2 | € 3 | 13 | -22% | -22% |  | 13 |
| Total expenditure for health services consumed by LHA inhabitants (E= A + B – C + D) | a104 | € 1.700 | € 1.682 | € 177 | 13 | 3% | 3% | 2% | 13 |

***Breakdown of LHA expenditure per areas of service with detail of general administrative costs***

| **Indicator** | **Code** | **Median** | **Average** | **Standard deviation** | **Number of answers** | **Median annual variation** | **Average annual variation** | **Standard deviation** | **Number of answers** |
| --- | --- | --- | --- | --- | --- | --- | --- | --- | --- |
| Hospital admissions | u1 | € 661 | € 697 | € 89 | 13 | 1% | 2% | 5% | 13 |
| Pharmaceuticals | c1 | € 251 | € 273 | € 37 | 13 | 2% | 2% | 4% | 13 |
| Outpatient specialist visits and exams | b1 | € 234 | € 220 | € 76 | 13 | 5% | 7% | 6% | 13 |
| General Pratictioners, Pediatricians and night service | efg101 | € 93 | € 103 | € 9 | 13 | 3% | 3% | 3% | 13 |
| Elderly People admissions in residential structures | m1 | € 69 | € 77 | € 39 | 13 | 4% | 4% | 5% | 13 |
| Mental healthcare | s1 | € 59 | € 59 | € 22 | 13 | 2% | 2% | 3% | 13 |
| Prevention, Public health and screening | ir101 | € 63 | € 56 | € 14 | 12 | 3% | 3% | 3% | 12 |
| Emergency services | q1 | € 48 | € 49 | € 26 | 12 | 4% | 6% | 8% | 12 |
| Rehabilitation | p1 | € 19 | € 28 | € 27 | 12 | 2% | 9% | 20% | 12 |
| Disabled people | n1 | € 28 | € 35 | € 24 | 12 | 5% | 9% | 14% | 12 |
| Prosthesis | d1 | € 26 | € 27 | € 11 | 12 | 5% | 4% | 7% | 12 |
| Home care | h26 | € 22 | € 22 | € 11 | 12 | 6% | 7% | 8% | 12 |
| Mother and child | l1 | € 18 | € 18 | € 11 | 12 | 3% | 3% | 4% | 12 |
| Dependences | t1 | € 15 | € 16 | € 4 | 12 | 3% | 17% | 53% | 12 |
| Hospice | o1 | € 2 | € 3 | € 3 | 11 | 4% | 6% | 5% | 11 |
| Total expenditure for health services consumed by LHA inhabitants | a107 | € 1.700 | € 1.682 | € 171 | 13 | 3% | 3% | 2% | 13 |

***Breakdown of outpatient specialist expenditure per type of producer***

| **Indicator** | **Code** | **Median** | **Average** | **Standard deviation** | **Number of answers** | **Median annual variation** | **Average annual variation** | **Standard deviation** | **Number of answers** |
| --- | --- | --- | --- | --- | --- | --- | --- | --- | --- |
| Outpatient specialist visits and exams produced by hospital structures managed by the LHA | b101 | € 158 | € 141 | € 77 | 11 | 0% | 4% | 8% | 11 |
| Outpatient specialist visits and exams produced by ambulatory structures managed by the LHA | b102 | € 34 | € 42 | € 26 | 10 | 0% | 4% | 11% | 10 |
| Outpatient specialist visits and exams purchased by other public and private suppliers | b103 | € 67 | € 82 | € 59 | 10 | 5% | 7% | 5% | 10 |
| Overall outpatient specialist visits and exams expenditure |  | € 231 | € 209 | € 76 | 11 | 2% | 4% | 6% | 11 |

***Breakdown of outpatient specialist visits and exams per type of activity***

| **Indicator** | **Code** | **Median** | **Average** | **Standard deviation** | **Number of answers** | **Median annual variation** | **Average annual variation** | **Standard deviation** | **Number of answers** |
| --- | --- | --- | --- | --- | --- | --- | --- | --- | --- |
| Overall number of laboratory exams consumed by LHA inhabitants (for 1.000 LHA inhabitants weighted for age and sex) | b106 | 15.858 | 17.015 | 5.602 | 11 | 6% | 3% | 11% | 11 |
| Overall number of diagnostic exams consumed by LHA inhabitants (for 1.000 LHA inhabitants weighted for age and sex) | b107 | 1.201 | 1.336 | 501 | 11 | 3% | 1% | 9% | 11 |
| Overall number of specialist visits consumed by LHA inhabitants (for 1.000 LHA inhabitants weighted for age and sex) | b108 | 6.140 | 11.725 | 10.869 | 11 | 6% | 4% | 12% | 11 |

General practitioners, paediatricians and night service

| **Indicator** | **Code** | **Median** | **Average** | **Standard deviation** | **Number of answers** | **Median annual variation** | **Average annual variation** | **Standard deviation** | **Number of answers** |
| --- | --- | --- | --- | --- | --- | --- | --- | --- | --- |
| Number of GPs per 1.000 inhabitants | e12 | 0,82 | 0,81 | 0,07 | 13 | -1% | -1% | 1% | 12 |
| % people assisted by GP in “groups” practices | e9 | 18% | 21% | 10% | 13 | 40% | 51% | 55% | 11 |
| % people assisted by GP in “network” practices | e10 | 16% | 20% | 13% | 13 | 38% | 58% | 76% | 10 |
| % people assisted by GP in “association” practices | e11 | 19% | 24% | 13% | 13 | -10% | -10% | 11% | 12 |
| Weekly number of hours in which GPs studies are open per 1.000 inhabitants | e19 | 11 | 10 | 3 | 13 | 5% | 5% | 4% | 11 |
| Number of GPs per 1.000 0-14 years inhabitants | f11 | 1,01 | 0,95 | 0,17 | 13 | 0% | 1% | 3% | 12 |
| % people assisted by Pediatricians in “groups” practices | f8 | 7% | 8% | 9% | 13 | 0% | 8% | 25% | 10 |
| % people assisted by Pediatricians in “network” practices | f9 | 0% | 1% | 2% | 11 |  |  |  | 10 |
| % people assisted by Pediatricians in “association” practices | f10 | 34% | 33% | 15% | 13 | 6% | 70% | 135% | 11 |
| Weekly number of hours in which Pediatricians studies are open per 1.0000-14 years inhabitants | f12 | 13 | 14 | 4 | 13 | 2% | 2% | 5% | 11 |
| Weekly number of hours in which 24 hours services are open per 1.000 inhabitants | g3 | 5 | 6 | 4 | 13 | 0% | 0% | 2% | 12 |
| Number of accesses to 24 hours services per 1.000 inhabitants | g4 | 37 | 56 | 57 | 12 | 2% | 5% | 9% | 11 |
| Number of home visits provided by night services per 1.000 inhabitants | g6 | 31 | 45 | 44 | 12 | -5% | -6% | 5% | 11 |

Home healthcare

| **Indicator** | **Code** | **Median** | **Average** | **Standard deviation** | **Number of answers** | **Median annual variation** | **Average annual variation** | **Standard deviation** | **Number of answers** |
| --- | --- | --- | --- | --- | --- | --- | --- | --- | --- |
| % people assisted on overall population | h1 | 2% | 3% | 2% | 12 | 5% | 5% | 7% | 12 |
| % people assisted on overall population > 65 years | h2 | 9% | 10% | 7% | 12 | 4% | 4% | 7% | 12 |
| Overall number of accesses to patients home per 1.000 inhabitants | h18 | 494 | 494 | 295 | 13 | 7% | 5% | 4% | 11 |

Mother and child services

| **Indicator** | **Code** | **Median** | **Average** | **Standard deviation** | **Number of answers** | **Median annual variation** | **Average annual variation** | **Standard deviation** | **Number of answers** |
| --- | --- | --- | --- | --- | --- | --- | --- | --- | --- |
| Number of accesses to mother and child care structures per 1.000 inhabitants | l15 | 143 | 174 | 128 | 12 | 6% | 6% | 12% | 12 |
| Number of assisted people per 1.000 inhabitants | l16 | 26 | 33 | 31 | 12 | 2% | 10% | 23% | 11 |
| ***Older people (>65)*** |  |  |  |  |  |  |  |  |  |
| **Indicator** | **Code** | **Median** | **Average** | **Standard deviation** | **Number of answers** | **Median annual variation** | **Average annual variation** | **Standard deviation** | **Number of answers** |
| Number of days in residential structures per 1.000 inhabitants > 65 years | m14 | 5.063 | 5.423 | 4.519 | 13 | 2% | 0% | 13% | 11 |
| Number of days in ambulatory structures per 1.000 inhabitants > 65 years | m15 | 214 | 396 | 482 | 13 | 10% | 18% | 27% | 12 |
| Number of days in community centers per 1.000 inhabitants > 65 years | m16 | - | 4 | 9 | 12 | 13% | 13% | 14% | 11 |
| % of people > 65 years admitted in residential structures per 1.000 inhabitants | m17 | 0% | 4% | 6% | 13 | 0% | 2% | 4% | 11 |

Disabled people

| **Indicator** | **Code** | **Median** | **Average** | **Standard deviation** | **Number of answers** | **Median annual variation** | **Average annual variation** | **Standard deviation** | **Number of answers** |
| --- | --- | --- | --- | --- | --- | --- | --- | --- | --- |
| Number of days in residential structures per 1.000 inhabitants | n7 | 113 | 115 | 82 | 12 | -2% | -1% | 6% | 11 |
| Number of days in ambulatory structures per 1.000 inhabitants | n101 | 93 | 97 | 82 | 11 | 5% | 6% | 5% | 11 |

Rehabilitation and long term care

| **Indicator** | **Code** | **Median** | **Average** | **Standard deviation** | **Number of answers** | **Median annual variation** | **Average annual variation** | **Standard deviation** | **Number of answers** |
| --- | --- | --- | --- | --- | --- | --- | --- | --- | --- |
| Number of days in rehabilitation structures per 1.000 inhabitants | p6 | 0 | 21 | 35 | 11 | -1% | 13% | 30% | 11 |
| Number of admissions in rehabilitation structures per 1.000 inhabitants | p7 | 3 | 24 | 47 | 12 | 0% | -6% | 20% | 10 |

Emergency services

| **Indicator** | **Code** | **Median** | **Average** | **Standard deviation** | **Number of answers** | **Median annual variation** | **Average annual variation** | **Standard deviation** | **Number of answers** |
| --- | --- | --- | --- | --- | --- | --- | --- | --- | --- |
| Number of accesses per 1.000 inhabitants weighted for age and sex | q9 | 339 | 318 | 134 | 12 | 1% | 1% | 2% | 9 |
| White codes per 1.000 inhabitants weighted for age and sex | q10 | 64 | 88 | 69 | 12 | -4% | 20% | 66% | 9 |
| White codes per 1.000 inhabitants > 65 years weighted for age and sex | q13 | 42 | 60 | 38 | 9 | 10% | 30% | 68% | 11 |
| White codes per 1.000 inhabitants > 75 years weighted for age and sex | q14 | 55 | 61 | 35 | 9 | 6% | 29% | 66% | 11 |
| White codes per 1.000 inhabitants > 90 years weighted for age and sex | q15 | 42 | 55 | 21 | 8 | -1% | 19% | 52% | 11 |

Prevention, public health and screening

| **Indicator** | **Code** | **Median** | **Average** | **Standard deviation** | **Number of answers** | **Median annual variation** | **Average annual variation** | **Standard deviation** | **Number of answers** |
| --- | --- | --- | --- | --- | --- | --- | --- | --- | --- |
| % compulsory vaccination for children | r3 | 97% | 86% | 29% | 13 | 0% | 0% | 1% | 10 |
| Number of visits for mobility allowance per 1.000 inhabitants | r7 | 9 | 13 | 10 | 12 | 3% | 6% | 8% | 9 |
| ***Mental health*** |  |  |  |  |  |  |  |  |  |
| **Indicator** | **Code** | **Median** | **Average** | **Standard deviation** | **Number of answers** | **Median annual variation** | **Average annual variation** | **Standard deviation** | **Number of answers** |
| Number of ambulatory visits per 1.000 inhabitants | s16 | 56 | 109 | 124 | 13 | 4% | 8% | 13% | 11 |
| Number of home visits per 1.000 inhabitants | s17 | 13 | 28 | 40 | 13 | 4% | 4% | 6% | 8 |
| Number of compulsory admissions per 1.000 inhabitants | s18 | 0,19 | 0,31 | 0,50 | 13 | -9% | -6% | 17% | 12 |
| Number of people taken in charge per 1.000 inhabitants | s23 | 11 | 9 | 6 | 13 | 4% | 3% | 3% | 10 |
| Number of accesses to Mental Health Services per 1.000 inhabitants | s25 | 192 | 182 | 152 | 13 | 8% | 15% | 28% | 11 |
| ***Dependencies*** |  |  |  |  |  |  |  |  |  |
| **Indicator** | **Code** | **Median** | **Average** | **Standard deviation** | **Number of answers** | **Median annual variation** | **Average annual variation** | **Standard deviation** | **Number of answers** |
| Number of days in residential structures per 1.000 inhabitants | t3 | 61 | 65 | 29 | 13 | -1% | 1% | 8% | 11 |

Hospital admissions

| **Indicator** | **Median** | **Average** | **Standard deviation** | **Number of answers** | **Median annual variation** | **Average annual variation** | **Standard deviation** | **Number of answers** |
| --- | --- | --- | --- | --- | --- | --- | --- | --- |
| Number of ordinary admissions in Hospitals managed by the LHA per 1.000 inhabitants weighted for age and sex | 63 | 55 | 33 | 9 | -3% | -3% | 3% | 9 |
| Number of ordinary admissions in Hospitals managed by other public and private hospitals per 1.000 inhabitants weighted for age and sex | 66 | 79 | 47 | 11 | -1% | -1% | 1% | 11 |
| Number of Day Hospitals admissions in Hospitals managed by the LHA per 1.000 inhabitants weighted for age and sex | 27 | 22 | 15 | 10 | -2% | -2% | 8% | 10 |
| Number of Day Hospitals admissions in Hospitals managed by other public and private hospitals per 1.000 inhabitants weighted for age and sex | 24 | 35 | 29 | 10 | 2% | -1% | 7% | 10 |
| Number of admissions with high risk of inappropriateness (51 DRGs) per 1.000 inhabitants weighted for age and sex | 17 | 18 | 13 | 13 | -5% | -6% | 5% | 13 |
| Average length of stay of admissions (number of days) | 8 | 8 | 2 | 13 | 0% | -1% | 5% | 11 |

**Appendix 2. CRITERIA USED TO WEIGHT THE INHABITANTS’ CONSUMPTION of OUTPATIENT VISITS to specialists AND EXAMinations, PHARMACEUTICALS AND HOSPITAL ADMISSIONS, BASED ON AGE AND SEX (Source: Italian Ministry of Health, “Criteri di riparto del Fondo Sanitario Nazionale”, 2007).**

| **AGE AND SEX** | **0** | **1-4** | **5-14** | **15-44 male** | **15-44 female** | **45-64** | **65-74** | **Over 75** |
| --- | --- | --- | --- | --- | --- | --- | --- | --- |
| Outpatient Specialist visits and exams | 0.242 | 0.204 | 0.169 | 0.228 | 0.363 | 0.573 | 1 | 0.987 |
| Pharmaceuticals | 1 | 0.969 | 0.695 | 0.693 | 0.771 | 2.104 | 4.176 | 4.290 |
| Hospital admissions | 2.539 | 0.376 | 0.254 | 0.392 | 0.567 | 0.945 | 2.105 | 3.025 |
